# Use of antithrombotics at the end of life: an in-depth chart review study

**DOI:** 10.1186/s12904-021-00786-3

**Published:** 2021-07-16

**Authors:** Bregje A.A. Huisman, Eric C.T. Geijteman, Jimmy J. Arevalo, Marianne K. Dees, Lia van Zuylen, Karolina M. Szadek, Agnes van der Heide, Monique A.H. Steegers

**Affiliations:** 1 Department of Anesthesiology, Amsterdam UMC, Vrije Universiteit Amsterdam, De Boelelaan 1117, 1081 HV Amsterdam, The Netherlands; 2 Hospice Kuria, Amsterdam, The Netherlands; 3 Department of Medical Oncology, Erasmus MC Cancer Institute, Rotterdam, The Netherlands; 4 Department of Public Health, Erasmus University Medical Center, Rotterdam, The Netherlands; 5 Department of IQ Healthcare, Radboud University Medical Center, Nijmegen, The Netherlands; 6 Department of Medical Oncology, Amsterdam UMC, Vrije Universiteit Amsterdam, Amsterdam, The Netherlands

**Keywords:** Palliative Care, End-of-life care, Bleeding, Anticoagulants, Venous thromboembolism

## Abstract

**Background:**

Antithrombotics are frequently prescribed for patients with a limited life expectancy. In the last phase of life, when treatment is primarily focused on optimizing patients’ quality of life, the use of antithrombotics should be reconsidered.

**Methods:**

We performed a secondary analysis of a retrospective review of 180 medical records of patients who had died of a malignant or non-malignant disease, at home, in a hospice or in a hospital, in the Netherlands. All medication prescriptions and clinical notes of patients using antithrombotics in the last three months of life were reviewed manually. We subsequently developed case vignettes based on a purposive sample, with variation in setting, age, gender, type of medication, and underlying disease.

**Results:**

In total 60% (*n*=108) of patients had used antithrombotics in the last three months of life. Of all patients using antithrombotics 33.3 % died at home, 21.3 % in a hospice and 45.4 % in a hospital. In total, 157 antithrombotic prescriptions were registered; 30 prescriptions of vitamin K antagonists, 60 of heparins, and 66 of platelet aggregation inhibitors. Of 51 patients using heparins, 32 only received a prophylactic dose. In 75.9 % of patients antithrombotics were continued until the last week before death. Case vignettes suggest that inability to swallow, bleeding complications or the dying phase were important factors in making decisions about the use of antithrombotics.

**Conclusions:**

Antithrombotics in patients with a life limiting disease are often continued until shortly before death. Clinical guidance may support physicians to reconsider (dis)continuation of antithrombotics and discuss this with the patient.

**Supplementary Information:**

The online version contains supplementary material available at 10.1186/s12904-021-00786-3.

## Background

Antithrombotics are frequently prescribed to patients with a life-limiting disease [1–7]. They mainly involve platelet aggregation inhibitors (PAIs) or anticoagulants. PAIs are mainly prescribed for primary or secondary prevention of arterial thrombosis, such as non-cardiogenic ischemic stroke [8], and for coronary and peripheral arterial disease (PAD) [9, 10]. Anticoagulants include three types: 1) heparins, 2) vitamin K antagonists (VKA), and 3) non-VKA oral anticoagulants (NOACs). The majority of patients with a life-limiting disease use anticoagulants for the prevention of thromboembolic events due to atrial fibrillation (AF) [11]. Other main reasons for using anticoagulants in the last phase of life are primary or secondary prevention and treatment (tertiary prevention) of venous thromboembolism (VTE) and prevention of thromboembolic events after heart valve replacement.

Most patients have been taking antithrombotics for a long period of time before their life expectancy becomes limited due to an illness. The number of patients still using antithrombotics at the end of life may vary depending on the population, setting and country. Of 16,896 patients with lung cancer who were admitted to a hospice, 9.2 % used an anticoagulant, of whom about 80 % used a VKA and the remaining 20 % a low-molecular-weight heparin (LMWH) [12]. In a retrospective study, 1141 patients who were admitted for acute care in a hospital were discharged to hospice care [11]. Of these patients, 64 % had a cardiovascular disease, and about 20 % suffered from cerebrovascular disease. Only 77 (6,7 %) patients had a prescription for antithrombotic therapy upon their discharge to hospice care. The main indications for antithrombotic use in this study were AF (41.6 %), history of stroke (31.2 %), deep venous thrombosis (DVT) or pulmonary embolism (PE) (11.7 %) or heart valve replacement (10.4 %). Various other studies have shown that up to half of all patients with an advanced illness and frail elderly patients continue to use antithrombotics in the last weeks of life [1–4]. A large cohort study in Sweden in older adults showed that antithrombotic use was similar (about 50 %) 12 months and one month before death. Most of these patients used a PAI (44 %), about 7 % a VKA and 2–5 % a LMWH [1]. About 20–30 % of patients in a hospice or frail elderly still use PAIs [5–7].

In the last phase of life, when treatment is primarily focused on optimizing patients’ quality of life, the use of antithrombotics should be reconsidered since the risk benefit ratio may change [13–17]. Clinically apparent VTE occurs in as many as 10 % of cancer patients [18], whereas almost 10 % of patients experience a clinically relevant bleeding at the end of life, with about one fifth of these bleedings contributing to death [15].

Comprehensive guidelines for (dis)continuation of antithrombotics in patients with a life-limiting disease are not available [19, 20]. The lack of evidence on outcomes of (dis)continuation of medication at the end of life and the complexity of clinical situations may lead to dilemmas in decision-making regarding antithrombotics. Moreover, insufficient awareness promotes continuation of potentially inappropriate medication in the last phase of life [21]. This may unnecessarily result in suboptimal quality of life and quality of death for patients with malignant and non-malignant life-limiting diseases. Thromboembolic as well as bleeding complications may lead to acute severe distressing situations, for patients, family and health care professionals.

In this paper, we report on a chart review study of the prescription of antithrombotics for patients with a life expectancy of less than three months staying at home, in a hospice or a hospital. The aim of this study was to gain more insight into management of antithrombotics in patients with a life-limiting disease and to conduct an in-depth analysis of the (dis)continuation of antithrombotics in patients in the last three months of life.

## Methods

### Design

 We conducted a secondary analysis of a retrospective chart review within the MEDIcation management in the LAST phase of life (MEDILAST) project. MEDILAST is a multicenter mixed methods research project with the aim of understanding current medication use in the last phase of life. The project was carried out by Erasmus University Medical Center in Rotterdam, Radboud University Medical Center in Nijmegen, and Amsterdam University Medical Center Vrije Universiteit in Amsterdam, all in the Netherlands. The study design, data collection and results of overall medication use in the last week of life have been described previously [22].

### Data collection

We collected a convenience sample of medical records of patients who had died at home, in a hospice or in a hospital. We aimed to include 60 medical records in each region of influence of the participating centers (Rotterdam, Nijmegen and Amsterdam). In each region 20 medical records were drawn from the home, hospice and hospital setting. We asked physicians to select their two or three most recent cases of patients who had died expectedly from a chronic condition. For each region, we randomly selected a number of family physician practices and approached them by telephone. In each region, the hospice setting was represented by a high care hospice, where a palliative care physician is at all times available for consultation. For the hospital setting, we included records from patients who had died in an academic or peripheral hospital, at a cardiology, geriatrics, neurology, oncology or pulmonology ward.

Following acceptance to participate in the study, three physician researchers (second, third and fourth author) visited the clinical wards and practices to collect the data. A structured electronic form (MS Access 2013) was used to retrieve the information from the medical records. Demographics included age at the time of death, place of death and primary and comorbid diagnoses. Information about medication use in the last three months of life was registered, which included medication generic names, administration routes, doses and start and stop dates. The medical records were manually screened for information about the decision making on medication use. The indication for the antithrombotic was derived from clinical notes as well as primary and secondary diagnoses. All methods were carried out in accordance with relevant guidelines and regulations for data collection from medical records [23]. All medication prescriptions of patients using antithrombotics were reviewed manually and of each patient using antithrombotics a case description was made including decision-making regarding the medication. Subsequently, case descriptions were selected by purposive sampling, with variation in setting, age, gender, medication, underlying disease and medical history and were rewritten as illustrative case vignettes.

 The Medical Ethics Review Committee of the Amsterdam University Medical Center Vrije Universiteit approved the study protocol (ref. no. 2013/47). Approval in all other participating centers was obtained from the board of directors or relevant authority before data collection.

### Data analysis

Medications were coded using the World Health Organization Center for Drug Statistics Methodology’s Anatomical Therapeutic Chemical (ATC) classification at the level of therapeutic subgroup (second), pharmacological subgroup (third), medication chemical subgroup (fourth) and chemical substance (fifth) [24]. IBM SPSS Statistics 26 (IBM Corporation, 2019) was used to obtain descriptive statistics.

## Results

A total of 180 medical records were reviewed in this study. One patient died within 24 h of admission to a hospice and was not included in the analysis. In the last three months of life, 60% (*n*=108) of patients had used antithrombotics. Table 1 shows the characteristics of patients using antithrombotics per setting. Of all patients using antithrombotics 46.3 % were female; 33.3 % died at home, 21.3 % in a hospice and 45.4 % in a hospital. The period of time for which medication use was registered ranged from one day to three months (mean 43.8 days, median 24 days). In total, 157 antithrombotic prescriptions were registered (see Table 2). In 82 patients (75.9 %), antithrombotics were continued until the last week before death. Case vignettes of patients using antithrombotics are presented in Tables 3, 4 and 5.

**Table 1:** Patient characteristics per setting

Characteristics	Setting			Total *n* = 108
	**Home care** ***n* = 36**	**Hospice** ***n* = 23**	**Hospital** ***n* = 49**	
Age, mean (SD)	77.7 (13.2)	73.4 (10.7)	74.8 (12.1)	75.5 (12.2)
Female gender, No. (%)	20 (55.6)	8 (34.8)	22 (44.9)	50 (46.3)
Primary diagnosis, No. (%) Cancer Cardiovascular disease Non-malignant lung disease Cerebrovascular disease Other	21 (58.3) 11 (30.6) 2 (5.6) 2 (5.6) 0	20 (87.0) 2 (8.7) 0 0 1 (4.3)	13 (26.5) 15 (30.6) 10 (20.4) 8 (16.3) 3 (6.1)	54 (50.0) 28 (25.9) 12 (11.1) 10 (9.3) 4 (3.7)

*SD* standard deviation

**Table 2:** Antithrombotic prescriptions

Antithrombotic agent		N=	%
VKA		30	19.1
	*phenprocoumon*	*1*	*0.6*
	*acenocoumarol*	*29*	*18.5*
Heparins		60	38.2
	*heparin*	*2*	*1.3*
	*dalteparin*	*8*	*5.1*
	*enoxaparin*	*7*	*4.5*
	*nadroparin*	*39*	*24.8*
	*tinzaparin*	*4*	*2.5*
PAI		66	42.0
	*clopidogrel*	*4*	*2.5*
	*dipyridamole*	*5*	*3.2*
	*acetylsalicylic acid*	*30*	*19.1*
	*carbasalate calcium*	*27*	*17.2*
Other	*fondaparinux*	*1*	*0.6*
	Total	157	100

*VKA* vitamin K antagonist, *PAI* platelet aggregation inhibitor

### Vitamin K antagonists

In total, 30 prescriptions of VKAs were registered in 29 patients, of whom 41.4 % died at home, 10.3 % in a hospice and 48.3 % in a hospital. Only one patient used phenprocoumon, all other prescriptions involved acenocoumarol (n = 29). In one patient acenocoumarol was stopped because of an intervention and restarted. The indications for the VKA varied from atrial fibrillation (Table 3, case 1 and 2), cardiac failure, cardiac valve disease, PE, DVT, to PAD (Table 3, case 3) and previous cerebrovascular accident (CVA) or transient ischemic attack (TIA) (Table 3, case 1). In total, 23 patients stopped using the VKA before death, of whom 17 patients less than one week before death (range 0–33 days, mean 5.2 days, median 2 days). In four patients bleeding complications were registered: painful hematoma in the flank (92 yrs, AF, TIA and urosepsis), fatal subdural hematoma (87 yrs, previous myocardial infarction, cardiac valve disease and renal insufficiency, combined with PAI), hematomas due to frequent falls (Table 3, case 2) and lung bleeding in lung cancer (Table 3, case 3). In some cases the VKA was stopped because of a prolonged INR (78 yrs, AF, cardiac failure, PAD and dialysis; 99 yrs, AF, cardiac failure and renal insufficiency). Other reasons for discontinuation that were mentioned were difficulty to swallow medication, drowsiness (Table 3, case 1), interaction with fluconazole, clinical deterioration, short life expectancy and patient’s request. In one patient suffering from an ileus the VKA was switched to therapeutic LMWH.

**Table 3:** Vitamin K antagonist case vignettes

Vitamin K antagonist
**1.** 82-year-old woman at home with pancreatic carcinoma. Atrial fibrillation, arterial hypertension, TIA, NIDDM **Acenocoumarol (VKA)** Comedication: amitriptyline retard 1dd 50 mg, amlodipine 1dd 5 mg, atorvastatin 10 mg, bisoprolol 1dd 2.5 mg, candesartan 1dd 16 mg, metformin 1dd 500 mg, pantoprazole 1dd 40 mg, temazepam 1dd 10 mg a.n. Seventeen days before death metformin is increased to 2dd 500 mg because of an increase in HbA1c. Ten days before death she is drowsy and nauseous. Metformin is stopped and glimepiride 1dd 2 mg is started. Amitriptyline, amlodipine and atorvastatin is stopped. The candesartan is decreased to 1dd 8 mg. Three days later she has a glucose of 1.8 mmol/L (32.4 mg/dL) and glimepiride is stopped. Three days later metformin is restarted at 2dd 250 mg because of a glucose of 8.6 mmol/l (154.8 mg/dL). She deteriorates, and three days later she is unconscious. All medication (including the acenocoumarol) is stopped and morphine suppository 4dd 20 mg is started. She dies the following day.
**2.** 95-year-old woman at home with arterial hypertension, atrial fibrillation, COPD and dementia. **Acenocoumarol (VKA)** Comedication: cholecalciferol 1dd 800 IE, furosemide 1dd 80 mg, digoxin 1dd 0.125 mg, losartan 1dd 25 mg, temazepam 1dd 10 mg a.n. She suffers from dyspnea and therefore the furosemide is increased with 40 mg/day extra for ten days. She frequently falls and suffers from hematomas. The acenocoumarol is stopped. Two days later she has fever and dyspnea. All other medication is stopped, she receives some morphine and midazolam and passes away.
**3.** 67-year-old man at home with metastatic lung carcinoma. Previous history of arterial hypertension, peripheral arterial disease and NIDDM. **Acenocoumarol (VKA)** Comedication: bisacodyl suppository 1dd 10 mg p.r.n., ibuprofen 1d 600 mg, metformin 1dd 500 mg, midazolam s.c. 15 mg p.r.n. (in case of lung bleeding), macrogol/electrolytes 1dd 1 sachet, oxycodone IR 6 dd 5 mg p.r.n., pantoprazole 1dd 40 mg, paracetamol 4dd 1000 mg, simvastatin 1dd 40 mg, zolpidem 1dd 10 mg a.n. p.r.n. Because of a pneumonia amoxicillin 3 dd 500 mg for a week and prednisone 3dd 10 mg for two weeks is started. He receives metoclopramide 3dd 10 mg rectally as needed because of vomiting due to a gastroenteritis. A month later he suffers from a dry cough and ipratropium inhaler 4dd and codeine 3dd 20 mg as needed is started, with limited effect. A week later he is admitted because of hemoptysis and the acenocoumarol is stopped. Back home oxazepam 1dd 10 mg as needed is started in case of anxiety. He gradually deteriorates and dies a month later.

*TIA* transient ischemic attack, *NIDDM* non-insulin dependent diabetes mellitus, *VKA* vitamin K antagonist, *COPD* chronic obstructive pulmonary disease, *a.n.* ante noctem, *p.r.n.* pro re nata (as needed), *s.c.* subcutaneous

### Heparins

In total, 60 prescriptions were registered in 51 patients, of whom 13.7 % died at home, 23.5 % in a hospice and 62.7 % in a hospital. Thirty-two patients received LMWH only in a prophylactic dose because of an acute medical condition or bedriddenness (Table 4, case 1). In three patients the prescribed heparin was changed to a different formulation. In two patients the dose was adjusted from a prophylactic dose to a therapeutic dose, because of newly diagnosed PE in one case and unknown condition in another case. In one patient with cardiac failure a heparin pump was discontinued, because of the decision to discontinue life-sustaining treatments, and switched to prophylactic LMWH. In several patients a heparin was started a few weeks before death (e.g. Table 4, case 1 and 2). Even in an unconscious or sedated patient the heparin was continued (Table 4, case 1, 2 and 3). A patient with colon carcinoma and previous PE received therapeutic dose LMWH until death, despite frequent nose bleeding. In total, 31 patients stopped using the heparins before death, of whom 21 patients less than one week before death (range 0–59 days, mean 5.5 days, median 1 day). Some specific reasons were mentioned for discontinuation. In two patients because of a bleeding (LMWH prophylaxis in laryngeal carcinoma and prophylaxis in a patient with an intracranial tumor) and once as family wanted unnecessary medication to be discontinued

**Table 4:** Heparins case vignettes

Heparins
**1.** 87-year-old woman with an intracerebral tumor and secondary epilepsy for which she receives comfort care. Further hypothyroidism and dyslipidemia. Acetylsalicylic acid 1dd 80 mg Comedication: dexamethasone 2dd 2 mg, phenytoin 2dd 150 mg, gemfibrozil 1dd 600 mg, levetiracetam 2dd 1000 mg, pantoprazole 1dd 40 mg, paracetamol 3dd 1000 mg p.r.n., temazepam 1dd 10 mg a.n., thyroxine 1dd 0.025 mg She is admitted at the hospital because of persisting partial convulsions. In between she is lucid and well-oriented. Clonazepam 2dd 0.5 mg is added and levetiracetam is gradually increased up to 2dd 1750 mg. Valproic acid 2dd 500 mg is added, and clonazepam increased up to 3dd 2 mg. Midazolam s.c. is titrated as needed 0.5-5 mg. **Nadroparin 1dd 2850 IE** is started six days after admission because of bedriddenness. She develops an expressive aphasia and agitation. Haloperidol 1 mg as needed is added. Two and a half weeks after admission she turns unconscious. All medication is continued until death.
**2.** 53-year-old man at home with pancreatic carcinoma and IDDM. Medication: amlodipine/valsartan 1dd 10/160 mg, diclofenac 2dd 75 mg, fentanyl transdermal 25 mcgr/h, haloperidol 2dd 1 mg, insulin pump s.c., omeprazole 1dd 40 mg, paracetamol 4dd 1000 mg, natriumlaurylsulfoacetate/natriumcitrate/sorbitol rectal p.r.n. Because of increasing pain diclofenac is increased to 3dd 75 mg and fentanyl nasal 50 mcg/dose as needed is added. After two weeks the fentanyl nasal is increased to 100 mcg/dose as needed. One week later **tinzaparin 1dd 18,000 IE** is started because of clinical signs of a deep venous thrombosis on the calf. Diclofenac and fentanyl is stopped and a morphine pump is started s.c. 5 mg/h and bolus 4 mg s.c. as needed. Amlodipine/valsartan is stopped because of low blood pressure. A week later palliative sedation is started with midazolam pump s.c. up to 15 mg/h. The oral medication is stopped, tinzaparin is continued. He passes away two days later.
**3.** 77-year-old woman with metastasized esophageal carcinoma and deep venous thrombosis is admitted to a hospice. Previous history: arterial hypertension, genital cancer, urinary tract infection. **Nadroparin (Fraxiparine forte) 1dd 15,200 IE** Comedication at admission: fentanyl transdermal 12 mcgr/h every 3 days, hydrochlorothiazide 1dd 12,5 mg, macrogol/electrolytes 1dd 1 sachet every two days, oxazepam 3dd 20 mg p.r.n., oxycodone IR 6dd 5 mg p.r.n., paracetamol 4dd 1000 mg p.r.n., temazepam 1dd 20 mg a.n. Two days after admission the hydrochlorothiazide is stopped, and haloperidol 1dd 1 mg and nitrofurantoin 4dd 50 mg is started because of signs of a delirium and cystitis. Fentanyl is switched to morphine retard 2dd 20 mg orally and morphine 5 mg 6dd orally as needed, because of pain and dyspnea. After two weeks morphine retard is increased to 2dd 30 mg because of dyspnea. After one week morphine 15 mg/24 h, haloperidol 2,5 mg/24 h and midazolam 42 mg/24 h is started s.c. because of refractory dyspnea and delirium. Oral medication is discontinued. She dies one day later. Nadroparin was continued until death.

*IDDM* insulin dependent diabetes mellitus, *a.n.* ante noctem, *p.r.n.* pro re nata (as needed), *s.c.* subcutaneous

### Platelet aggregation inhibitors

In total, 66 prescriptions of platelet aggregation inhibitors were registered in 55 patients, of whom 36.4 % died at home, 18.2 % at a hospice and 45.5 % in a hospital. Two of these patients used two PAIs (i.e. acetylsalicylic acid and clopidogrel). Other patients used a PAI and LMWH (n = 11) or VKA (n = 5), or two PAIs and a LMWH (n = 3) or VKA (n = 2). In three patients a PAI was switched to a different formulation in the last weeks of life. A patient suffering from sepsis, and previous history CVA, PAD, rheumatoid arthritis, diabetes and hypertension, used acetylsalicylic acid, clopidogrel and fondaparinux until start of palliative sedation. In total, 39 patients stopped using the PAI before death, of whom 29 patients less than one week before death (range 0–76 days, mean 5.9 days, median 1 day). In several patients the PAI was discontinued because of refusal by the patient, difficulty (Table 5, case 2) or discomfort to swallow medication. A 83-year old patient with a previous ischemic CVA and hypertension used acetylsalicylic acid and dipyridamole and died of a massive intracranial bleeding. In some patients the PAI was continued despite discontinuation of other oral preventive medications (Table 5, case 1). Table 5 case 3 presents a case vignette of a patient with lung cancer with cerebral metastases using a PAI because of intermittent claudication, in which the PAI is continued until death.

**Table 5:** Platelet aggregation inhibitor case vignettes

Platelet aggregation inhibitors
**1.** 86-year-old woman at home with heart failure, previous myocardial infarction, rectal blood loss and rheumatoid arthritis. **Carbasalate calcium 1dd 100 mg** Comedication: atenolol 1dd 100 mg, lisinopril 1dd 20 mg, loperamide 2dd 2 mg p.r.n., paracetamol 3dd 1000 mg, simvastatin 1dd 40 mg She receives nitrofurantoin 4dd 50 mg for five days because of a urinary tract infection. A month later she develops a pneumonia for which she receives doxycycline 1dd 100 mg for a week. After three days amoxicillin/clavulanic acid 3dd 500/125 mg is started for five days because of clinical deterioration. With the patient and her daughter it is decided not to admit to the hospital in case of further deterioration. She gradually deteriorates, has difficulty to swallow the medication. Atenolol, lisinopril, loperamide and simvastatin are stopped. She receives haloperidol droplets if needed in case of agitation. She passes away after more than a month. The paracetamol and carbasalate calcium were continued until death.
**2.** 87-year-old man at home with leukemia. Previous myocardial infarction, prostate carcinoma and NIDDM. **Acetylsalicylic acid 1dd 80 mg** Comedication: citalopram 1dd 20 mg, diltiazem 1dd 300 mg, esomeprazole 1dd 40 mg, furosemide 1dd 40 mg He gradually deteriorates. Oxazepam as needed 2dd 5 mg is started because of agitation two and half months before death. Two months later paracetamol suppository as needed 1000 mg and fentanyl 12 mcg/h transdermal is started because of pain. He is unable to swallow his medication and so all oral medication is stopped. Morphine s.c. as needed is started for pain and dyspnea. He passes away 6 days later.
**3.** 66-year-old man admitted to a hospice because of lung carcinoma with cerebral metastases. Previous history of intermittent claudication, colon carcinoma and benign prostate hypertrophy. **Carbasalate calcium 1dd 100 mg** Comedication at admission: dexamethasone 1dd 3 mg, esomeprazole 1dd 40 mg, macrogol/electrolytes 1dd 1 p.r.n., metformin 2dd 1000 mg, paracetamol 4dd 1000 mg p.r.n., spironolactone 1dd 25 mg As needed midazolam and morphine s.c. is ordered in case of massive hemoptysis or brain herniation. After a week the dexamethasone is increased because of headache and nausea. Insulin is given in case of high blood glucose levels. Symptoms decrease and in a month the dexamethasone can be decreased to 1dd 1 mg. However, a few days later symptoms recur, dexamethasone is increased to 1dd 3 mg and after 2.5 weeks to 1dd 8 mg. A month later he further deteriorates. He receives morphine s.c. 5 mg as needed for dyspnea and pain and passes away two days later. All oral medication was continued until death.

*NIDDM *non-insulin dependent diabetes mellitus, *p.r.n.* pro re nata (as needed), *s.c.* subcutaneous

## Discussion

In this chart review study we found that a large number of patients used antithrombotics in the last three months, weeks or days of their lives. PAIs and heparins were more frequently prescribed than VKA, and antithrombotic prescriptions were more frequent in patients who died in a hospital. The case vignettes reveal examples of what happens in clinical practice on the patient level and show that antithrombotics are continued until death, irrespective of (dis)continuation of other medications in the last weeks of life. Antithrombotics may be of questionable benefit due to changes in goals of care in the last phase of life and the benefit-harm balance of this medication.

In our study, we found a relatively high prevalence of antithrombotic use (60 %) compared to previous studies [1–3, 5]. We do not have a plausible explanation for this. As in the other studies, the antithrombotics in our study mainly involved PAIs. The second most frequently used antithrombotic in patients with a life-limiting disease is VKAs or LMHWs depending on the population studied [1, 4, 5, 12]. In our study a relatively high number of patients used LMWHs, with about half of the prescriptions concerning a prophylactic dose.

### Dilemmas in decision-making

We hypothesize that physicians may be reluctant to discontinue antithrombotics due to complex clinical dilemmas. On the one hand, the medical assessment of potential benefits of (dis)continuing antithrombotics and minimizing the risk of adverse events is complicated and hampered by uncertainties. On the other hand, it is a challenge to discuss the use of antithrombotics with the patient and to reach a shared decision, taking into account benefits versus risks, patients’ quality of life and perceived medication burden. Studies into dilemmas in decision-making regarding antithrombotics in patients with a limited life expectancy, mostly concern anticoagulation in cancer patients and deprescribing of medication in frail elderly. Dilemmas in decision-making regarding antithrombotics in patients with a limited life expectancy reflected by the case vignettes and described in literature are multiple [25–32]. Examples of these dilemmas are: when to (dis)continue anticoagulants in patients with AF and cancer (Table 3, case 1), old age, frailty (Table 3, case 2) or risk of falls; when to (dis)continue PAIs in patients with cardiovascular disease combined with or without cancer (Table 5); when (not) to start or stop thromboprophylaxis (e.g. bedriddenness) (Table 4, case 1); when (not) to start or stop anticoagulants in cancer patients with symptomatic or incidental VTE (Table 4, case 2 and 3); how to balance the risk of bleeding versus PE in patients with lung carcinoma (Table 3, case 3); how to manage antithrombotics in patients with an increased risk of bleeding due to comorbidity (e.g. thrombocytopenia); which PAI or (dose of) anticoagulant is most appropriate.

### Cancer associated thrombosis

Noble and colleagues especially contributed to this field with their studies into cancer associated thrombosis (CAT) and mention that according to guidelines indefinite anticoagulation should be considered for patients with cancer and VTE [33–35]. Particularly, stomach and pancreas carcinoma are associated with a very high risk of CAT, and lymphoma, lung, gynecologic, bladder, testicular and renal carcinoma with a high risk [36]. However, though the majority of CAT patients with metastatic disease remain anticoagulated up to or within days of death, it appears that anticoagulation in this situation confers a significant bleeding risk without additional benefit of preventing VTE symptoms [33]. The OncPal deprescribing guideline is the only guideline for deprescribing of medication in oncological patients, and merely addresses the use of aspirin with the advice to discontinue it in case of primary prevention [37].

### (De)prescribing in elderly

Guidelines or tools for medication management in patients with a limited life expectancy due to non-malignant disease mainly involve those for elderly with advanced age, dementia or multimorbidity [38–40]. The Beers criteria and STOPP/START are some of the frequently used tools. The Beers criteria regarding antithrombotics advice to use aspirin for primary prevention with caution in adults above 70, prasugrel with caution above 75 years; to avoid oral short acting dipyridamole; and prescribe dabigatran and rivaroxaban for treatment of VTE or AF with caution in adults above 75 years; and avoid or reduce the dose of NOACs in the case of reduced renal function [41]. The STOPP/START give recommendations for adults above 65 years, mainly to avoid any antithrombotic in case of a significant bleeding risk; combine aspirin and clopidogrel only in selected cases; not to combine PAIs with VKA or NOACs; to discontinue VKA or NOACs after more than 6 respectively 12 months in patients without continuing provoking risk factors suffering a first DVT or PE [42]. Unfortunately, there is still a lack of standardization in assessment of medication appropriateness in patients with a limited life expectancy and a need for an approach that is specifically designed and validated for populations with a life-limiting illness [39].

### Need for clinical guidance

In this chart review study, we found that discontinuation of antithrombotics seems to be done mainly in reaction to a bleeding, inability to swallow or patient’s request. In a previous study we found that there is substantial variation in physicians’ opinions regarding the use of anticoagulants in patients with a limited life expectancy and they lack evidence about the risks and benefits of stopping anticoagulants in decision-making [43]. Which decision physicians eventually made seemed largely dependent on the choice of the patient [43]. Physicians need more evidence-based guidance to proactively discuss the use of antithrombotics and direction to come to a decision consistent with the patient’s goals and preferences. Patients with a life-limiting disease often prefer to reduce the number of medications they have to take, but they have concerns about the negative consequences or uncertainty of stopping the medication [44], risk of bleeding or thromboembolic complications [45]. Thus, clinical guidance may facilitate more rational and patient centered decisions. Therefore, we propose that a first step towards clinical guidance could be considered in practice to guide medical advice for antithrombotics and shared decision-making in patients with a life expectancy of less than three months (Supplement, with Figs. 1 and 2).

#### Limitations

 Our study involved a secondary analysis of data from a retrospective chart review study. Results might be influenced by selection bias and discrepancy between the registered prescriptions and actual use. Not in all cases the decision-making process was clear due to insufficient description in the clinical notes. Whereas the indication of antithrombotic use is not explicitly recorded in medical records, in most cases it was derived from clinical notes as well as patients’ primary and secondary diagnoses. Further, possibly relevant comorbid conditions, like renal function and coagulation disorders, were often not specifically registered with laboratory results, especially in patients in hospices or at home. Our sample of patients is relatively out of date concerning the use of NOACs. None of the patients used a NOAC. Specific considerations regarding NOACs were not represented, such as the relative ease in use or inferior efficacy in certain patient populations. However, the dilemmas regarding (dis)continuation are considered to be comparable to VKAs and therapeutic dose LMWHs. It was not the scope of this study to elaborate on the choice of anticoagulant for therapeutic use. Guidelines for the management of AF [46], VTE [47] and CAT [48] do give recommendations on the choice of anticoagulant, and many studies focus on this in complex patients with CAT [29, 49], AF in cancer patients [50] and in elderly patients [41, 51].

## Conclusions

A large number of patients with a life limiting disease still use antithrombotics in the last three months and even days of their lives. PAIs and heparins were more frequently prescribed than VKA, and antithrombotic prescriptions were more frequent in hospitalized patients. Dilemmas in decision-making regarding antithrombotics in patients with a limited life expectancy are reflected by case vignettes, and it seems that decision-making regarding antithrombotics is mainly a reactive process. The impact of possible thromboembolic and bleeding complications on quality of life and quality of death demand for a proactive decision-making regarding antithrombotics. Clinical guidance may support physicians to proactively give medical advice regarding (dis)continuation of antithrombotics and consecutively have a shared-decision making discussion with the patient.

## Supplementary Information

**Additional file 1.**

## Data Availability

The datasets generated and analyzed during the current study are not publicly available due to privacy of respondents, but are available from the corresponding author on reasonable request.

## Abbreviations

PAI: Platelet Aggregation Inhibitor
PAD: Peripheral Arterial Disease
VKA: Vitamin K antagonist
NOAC: Non-VKA Oral AntiCoagulant
AF: Atrial Fibrillation
VTE: Venous ThromboEmbolism
LMWH: Low-Molecular-Weight Heparin
DVT: Deep Venous Thrombosis
PE: Pulmonary Embolism
MEDILAST: MEDIcation management in the LAST phase of life
ATC: Anatomical Therapeutic Chemical
CVA: CerebroVascular Accident
TIA: Transient Ischemic Attack
(N)IDDM: (Non) Insulin Dependent Diabetes Mellitus
COPD: Chronic Obstructive Pulmonary Disease
CAT: Cancer Associated Thrombosis
