# Supplementary material for: Use of antithrombotics at the end of life: an in-depth chart review study
Source: BMC Palliat Care. 2021 Jul 16;20:110. doi: 10.1186/s12904-021-00786-3 (PMC8285840; doi:10.1186/s12904-021-00786-3)
Supplement: Supplementary file 1 — Additional file 1. [file 12904_2021_786_MOESM1_ESM.zip › 12904_2021_786_MOESM1_ESM_def_ESM.docx]

**Supplement: A first step towards clinical guidance**

We propose a first step towards clinical guidance that could be considered in practice in patients at home or staying in a health care facility to guide medical advice for antithrombotics (Figure 1); complementing a process of shared decision-making (Figure 2) in patients with a life expectancy of less than three months. Firstly, in patients with a life expectancy of less than three months any antithrombotic should be discontinued or not started in case of an active or recent troublesome bleeding, thus mentioned as a first consideration (Figure 1). Secondly, if the patient is in such a poor condition that the prognosis is very limited to days, medication should be reduced to the minimum to relieve suffering. Subsequently, in antithrombotic prescribing the balance between risks of thromboembolism and bleeding should be made. We intended to represent this as simple as possible, per group of antithrombotic, for PAIs, anticoagulants or the combination of both. We found guidelines regarding perioperative thrombotic risk stratification (1, 2) as a basic scheme for stratification as these take into account all cardiovascular disease. Thereafter this scheme was supplemented with recommendations from the CHA2DS2-VASc score (3), recent guidelines for the management of AF (4), VTE (5) and CAT (6); the Beers criteria (7), START/STOPP (8), the Khorana score (9) and the algorithm for VTE prevention in frail elderly patients with AF (10). Only in the case of a recent coronary stent, acute coronary syndrome (ACS) or high-grade symptomatic carotid stenosis a combination therapy could be appropriate. PAIs for primary prevention or low risk secondary prevention could be discontinued without significant risk. PAIs for high risk secondary prevention could be continued taking into account the patients preferences (see Figure 2). For anticoagulants in patients with a low to moderate thromboembolic risk, like isolated AF with CHA2DS2-VASc score of 0 to 7, the anticoagulant could be stopped. In patients with a high thromboembolic risk (>10%) further decision-making should be based on the bleeding risk. Therefore, the HAS-BLED (11) is suggested to determine the bleeding risk. In case of a high bleeding risk the anticoagulant should be discontinued, and with a low risk it could be continued taking into account the patients preferences (see Figure 2). The guidance for shared decision-making about antithrombotics at the end of life (Figure 2) is based on several studies into medication management in patients with a life-limiting illness in general and specifically concerning antithrombotics (12-15). The patient should be informed about the medical advice regarding the antithrombotic use (see Figure 1) and subsequently the patients preferences should be explored. Studies confirm that patients with a life-limiting disease wish to be involved in the decision-making process regarding antithrombotics (16, 17). Physicians should discuss the medication-related burden and what is important to the patient in terms of quality of life. Hypothetical scenarios could be helpful to discuss the fear of thromboembolic or bleeding complications and patients’ end-of-life care preferences (18). We would like to emphasize that management of antithrombotics is part of optimal palliative care aimed at quality of life and dying, and the importance of reassurance by discussing the options to relieve symptoms in case of certain disease or death scenarios. Both medical advice and shared decision-making should be reassessed periodically, for example, in the event of changes in the patient's clinical condition or transfer between settings.

**References**

1. Douketis JD, Lip GYH. Perioperative management of patients receiving anticoagulants. In: Leung LLK, Tirnauer JS, editors. UpToDate. Waltham, MA: UpToDate Inc.; Accessed October 7, 2020.

2. Mehdi Z, Birns J, Partridge J, Bhalla A, Dhesi J. Perioperative management of adult patients with a history of stroke or transient ischaemic attack undergoing elective non-cardiac surgery. Clin Med (Lond). 2016;16(6):535-40.

3. Lip GY, Nieuwlaat R, Pisters R, Lane DA, Crijns HJ. Refining clinical risk stratification for predicting stroke and thromboembolism in atrial fibrillation using a novel risk factor-based approach: the euro heart survey on atrial fibrillation. Chest. 2010;137(2):263-72.

4. Hindricks G, Potpara T, Dagres N, Arbelo E, Bax JJ, Blomstrom-Lundqvist C, et al. 2020 ESC Guidelines for the diagnosis and management of atrial fibrillation developed in collaboration with the European Association of Cardio-Thoracic Surgery (EACTS). European heart journal. 2020.

5. Lip GYH, Hull RD. Rationale and indications for indefinite anticoagulation in patients with venous thromboembolism. In: Mandel J, Leung LLK, editors. UpToDate. Waltham, MA: UpToDate Inc.; Accessed October 7, 2020.

6. Key NS, Khorana AA, Kuderer NM, Bohlke K, Lee AYY, Arcelus JI, et al. Venous Thromboembolism Prophylaxis and Treatment in Patients With Cancer: ASCO Clinical Practice Guideline Update. Journal of clinical oncology : official journal of the American Society of Clinical Oncology. 2020;38(5):496-520.

7. By the American Geriatrics Society Beers Criteria Update Expert P. American Geriatrics Society 2019 Updated AGS Beers Criteria® for Potentially Inappropriate Medication Use in Older Adults. J Am Geriatr Soc. 2019;67(4):674-94.

8. O'Mahony D, O'Sullivan D, Byrne S, O'Connor MN, Ryan C, Gallagher P. STOPP/START criteria for potentially inappropriate prescribing in older people: version 2. Age Ageing. 2015;44(2):213-8.

9. Khorana AA, Kuderer NM, Culakova E, Lyman GH, Francis CW. Development and validation of a predictive model for chemotherapy-associated thrombosis. Blood. 2008;111(10):4902-7.

10. Granziera S, Cohen AT, Nante G, Manzato E, Sergi G. Thromboembolic prevention in frail elderly patients with atrial fibrillation: a practical algorithm. J Am Med Dir Assoc. 2015;16(5):358-64.

11. Lip GY, Frison L, Halperin JL, Lane DA. Comparative validation of a novel risk score for predicting bleeding risk in anticoagulated patients with atrial fibrillation: the HAS-BLED (Hypertension, Abnormal Renal/Liver Function, Stroke, Bleeding History or Predisposition, Labile INR, Elderly, Drugs/Alcohol Concomitantly) score. Journal of the American College of Cardiology. 2011;57(2):173-80.

12. Hutchinson A, Rees S, Young A, Maraveyas A, Date K, Johnson MJ. Oral anticoagulation is preferable to injected, but only if it is safe and effective: An interview study of patient and carer experience of oral and injected anticoagulant therapy for cancer-associated thrombosis in the select-d trial. Palliat Med. 2019;33(5):510-7.

13. Dees MK, Geijteman ECT, Dekkers WJM, Huisman BAA, Perez R, van Zuylen L, et al. Perspectives of patients, close relatives, nurses, and physicians on end-of-life medication management. Palliat Support Care. 2018;16(5):580-9.

14. Sand AM, Harris J, Rosland JH. Living with advanced cancer and short life expectancy: patients' experiences with managing medication. J Palliat Care. 2009;25(2):85-91.

15. Voogt E, van der Heide A, Rietjens JA, van Leeuwen AF, Visser AP, van der Rijt CC, et al. Attitudes of patients with incurable cancer toward medical treatment in the last phase of life. Journal of clinical oncology : official journal of the American Society of Clinical Oncology. 2005;23(9):2012-9.

16. Zabrocka E, Wojtukiewicz MZ, Sierko E. Thromboprophylaxis in cancer patients in hospice. Advances in clinical and experimental medicine : official organ Wroclaw Medical University. 2018;27(2):283-9.

17. Delluc A, Wang TF, Yap ES, Ay C, Schaefer J, Carrier M, et al. Anticoagulation of cancer patients with non-valvular atrial fibrillation receiving chemotherapy: Guidance from the SSC of the ISTH. Journal of thrombosis and haemostasis : JTH. 2019;17(8):1247-52.

18. Dassel KB, Utz R, Supiano K, McGee N, Latimer S. The Influence of Hypothetical Death Scenarios on Multidimensional End-of-Life Care Preferences. Am J Hosp Palliat Care. 2018;35(1):52-9.

### **Figure legends**

### **Fig. 1. Preliminary clinical guidance for antithrombotic management at the end of life**

ACS: acute coronary syndrome, AF: atrial fibrillation, BMS: bare metal stent, CVA: cerebrovascular accident, DES: drug-eluting stent, DVT: deep venous thrombosis, LVEF: left ventricular ejection fraction, PCI: percutaneous coronary intervention, TE: thromboembolic, TIA: transient ischemic attack.

### **Fig. 2**. **Shared decision-making about antithrombotics at the end of life**

Guidance for discussion with the patient and/or relative(s). CVA: cerebrovascular accident, DVT: deep venous thrombosis, INR: international normalized ratio, LMWH: low-molecular-weight heparin.
