# Supplementary figures and images for: Use of antithrombotics at the end of life: an in-depth chart review study
Source: BMC Palliat Care. 2021 Jul 16;20:110. doi: 10.1186/s12904-021-00786-3 (PMC8285840; doi:10.1186/s12904-021-00786-3)

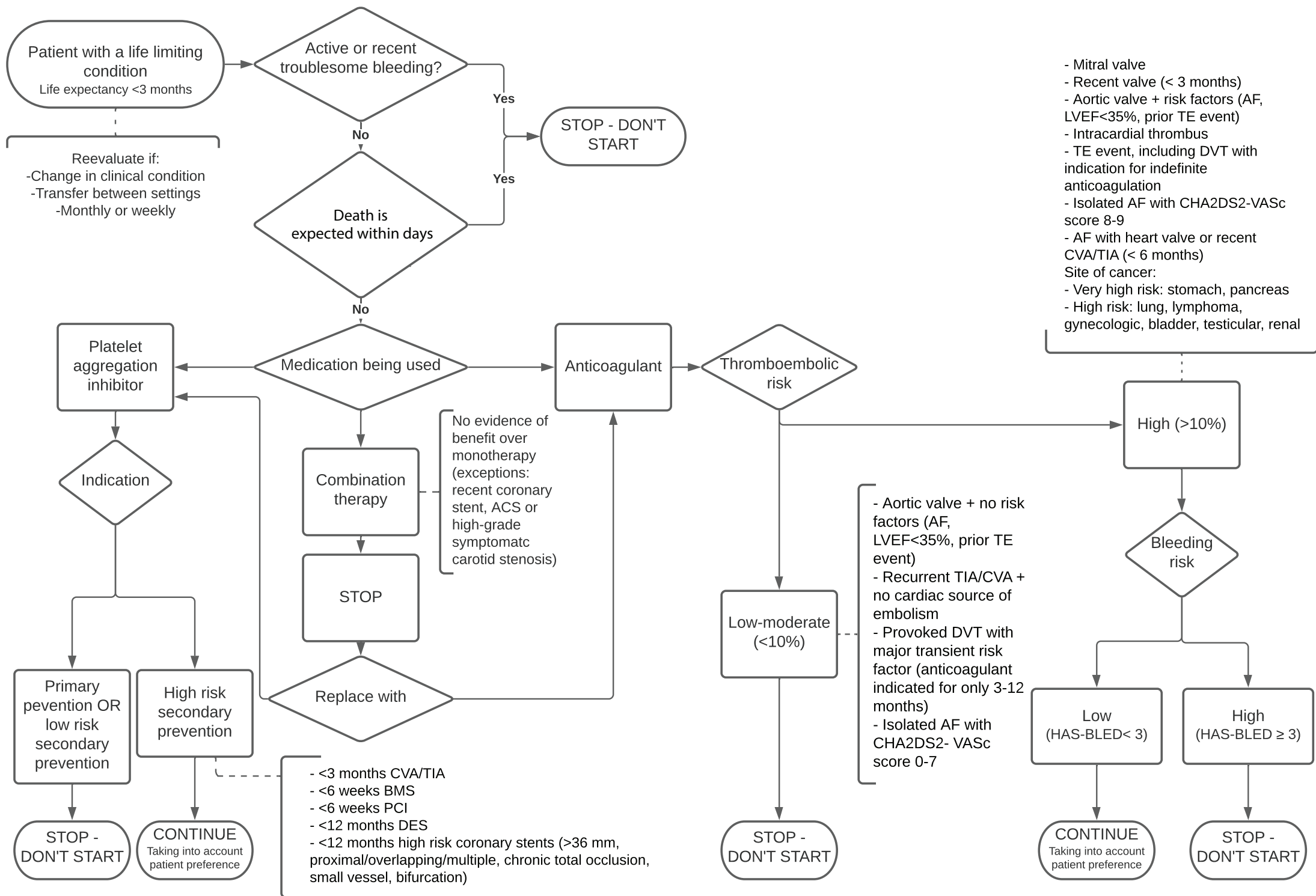

Supplement: Supplementary file 1 — Additional file 1. [file 12904_2021_786_MOESM1_ESM.zip › Figure 1_ESM.pdf]

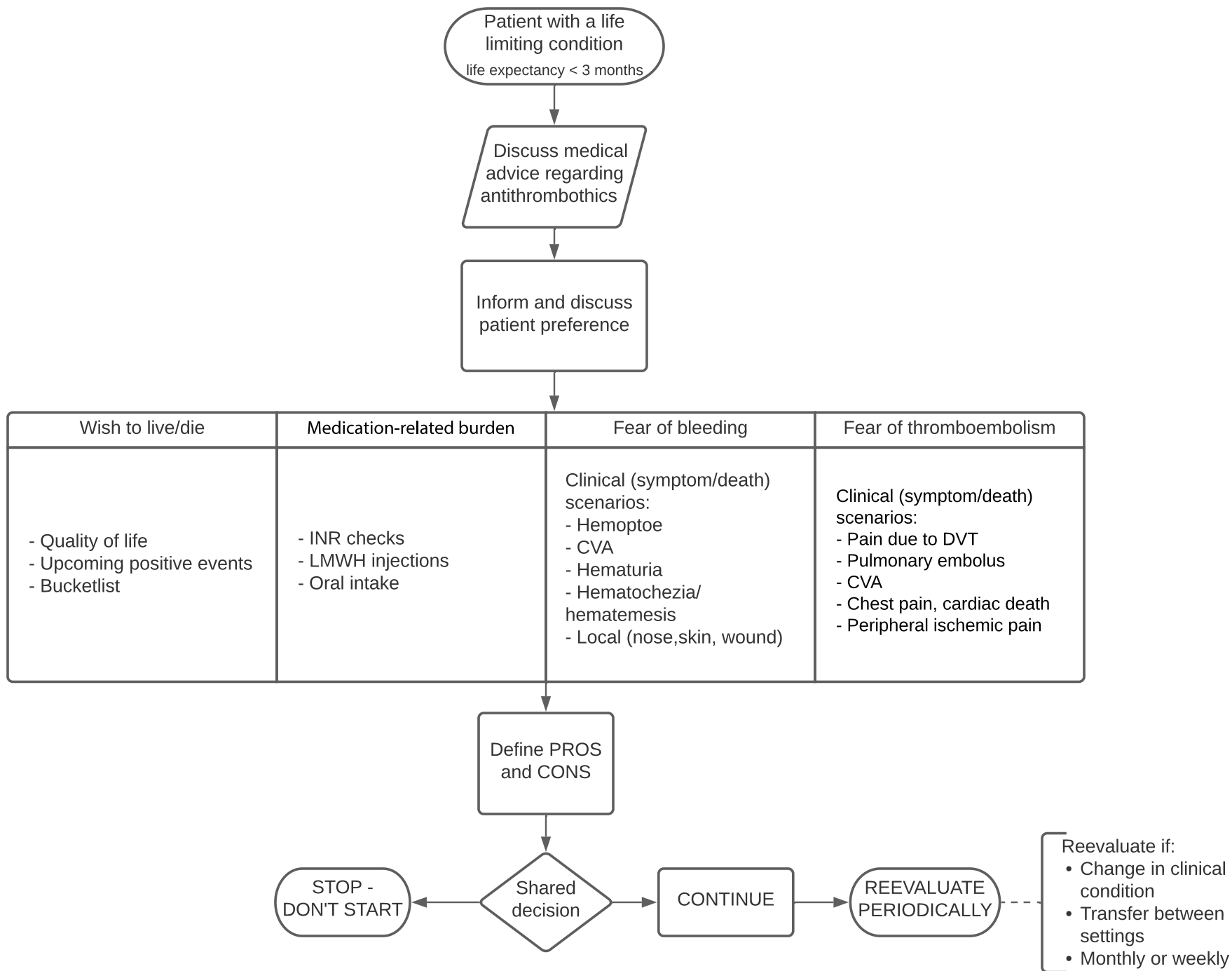

Supplement: Supplementary file 1 — Additional file 1. [file 12904_2021_786_MOESM1_ESM.zip › Figure 2_ESM.pdf]
